# A Prospective Cohort Study of AIS Patients with 40° and More Treated with a Gensingen Brace (GBW): Preliminary Results

**DOI:** 10.2174/1874325001711011558

**Published:** 2017-12-29

**Authors:** Hans-Rudolf Weiss, Nicos Tournavitis, Sarah Seibel, Alexander Kleban

**Affiliations:** 1 Gesundheitsforum Nahetal, Alzeyer Str. 23, D-55457 Gensingen, Germany; 2 Scoliosis Best Practice Rehab Services, Aristotelous 5, GR 54624, Thessaloniki, Greece; 3 Lomonosov Moscow State University, 119234, Leninskie Gory 1, Moscow, Russia

**Keywords:** Scoliosis, Brace treatment, BrAIST, Cheneau brace, Gensingen brace, Lumbar spine

## Abstract

**Introduction:**

There is a growing resistance from patients and their families to spinal fusion surgery for scoliosis. Due to inconclusive evidence that surgery has a long-term effect on scoliosis and/or improves the quality of life for patients with scoliosis, there is a need to extend the conservative perspective of treatment to patients with curvatures greater than 40 degrees. For that reason, a prospective cohort study was initiated to determine the effectiveness of the Gensingen brace (a Cheneau-style TLSO) in preventing progression in skeletally immature patients.

**Materials and Methods:**

Since 2011, fifty-five patients have been enrolled in this prospective cohort study. This report includes the mid-term results of twenty-five of these patients, who have a minimum follow-up of 18 months and an average follow-up of 30.4 months (SD 9.2). The twenty-five patients had the following characteristics at the start of treatment: Cobb angle: 49° (SD 8.4; 40º-71º); 12.4 years old (SD 0.82); Risser: 0.84 (SD 0.94; 0-2). A z-test was used to compare the success rate in this cohort to the success rate in the prospective braced cohort from BrAIST.

**Results:**

After follow-up, the average Cobb angle was 44.2° (SD 12.9). Two patients progressed, 12 patients were able to achieve halted progression, and eleven patients improved. Angle of trunk rotation (ATR) decreased from 12.2 to 10.1 degrees in the thoracic spine (p = 0.11) while the ATR decreased from 4.7 to 3.6 degrees in the lumbar spine (p = 0.0074). When comparing the success rate of the BrAIST cohort with the success rate of patients in this cohort, the difference was statistically significant (z = -3.041; p = 0.01).

**Conclusion:**

Conservative brace treatment using the Gensingen brace was successful in 92% of cases of patients with AIS of 40 degrees and higher. This is a significant improvement compared to the results attained in the BrAIST study (72%). Reduction of the ATR shows that postural improvement is also possible.

## 1 INTRODUCTION

Scoliosis is a three-dimensional deformity of the spine and trunk, which may deteriorate quickly during phases of rapid growth [1, 2]. The Cobb angle [3] determines the degree of lateral curvature on an x-ray while the angle of trunk rotation (ATR), as measured by Scoliometer ^TM^ allows for clinical evaluation and follow-up for patients with scoliosis [4].

Scoliosis has various etiologies (congenital, neuromuscular, mesenchymal disorders and others) [5]. However, Adolescent Idiopathic Scoliosis (AIS) [1, 2, 6], the most prevalent form, has an undetermined etiology and affects 80 – 90% of the patient population. Recently, MRI studies show signs of a functional tethering of the spinal cord [7], which may, in some cases, explain the thoracic flatback deformity, and ventral overgrowth [8] of the spinal column in this condition.

Treatment indications for scoliosis continue to be under debate [9, 10]. Conservative treatment of scoliosis, both rehabilitative exercise and bracing, are recognized in literature reviews [11-14], Cochrane reviews [15, 16] and randomized controlled studies [17-19]. Often, when a scoliosis reaches 45- 50º, surgery is the typical mode of treatment despite the absence of high-quality evidence [9, 14, 20-25]. Comprehensive reviews [11, 14, 20, 21, 23] and two Cochrane reviews [23, 24] failed to establish evidence supporting the position that surgery is superior to conservative treatment and/or to natural history [1, 2]. Recent comprehensive reviews show that the long-term risks of spinal fusion surgery are significant [14, 20, 26, 27].

While AIS can be a progressive condition and requires monitoring and management, it is a relatively benign disorder, which rarely leads to severe health consequences [28, 29]. Recently Ward *et al*. [30, 31] have demonstrated that spinal fusion surgery does not significantly improve Health-Related Quality of Life (HRQoL). They concluded, “This data in conjunction with an absence of long-term evidence of serious medical consequences with non-surgical management of curves ≥ 40° should encourage surgeons to reevaluate the benefits of routine surgical care [31].”

In consideration of these recent findings and the growing number of reviews supporting conservative treatments, the current indications for bracing should be re-evaluated and possibly expanded, when appropriate. Thus, it is important to research the efficacy of bracing for adolescent patients with scoliosis over 40°. With respect to bracing, it has been shown that the percentage of in-brace correction and brace-wearing time can have an effect on the eventual outcome of brace treatment [32]. That being said, each type of brace should be evaluated independently due to disparate results for different types of braces [33-45]. Independent studies do not show evidence in support of soft braces [33-35], however, strong evidence exists in support of rigid bracing [32, 33, 36, 37]. In Europe, a prospective study using a cast-based asymmetric Chêneau brace had a success rate of 80% [33]. Recent retrospective studies, also on the Chêneau brace, demonstrate success rates of more than 90% [38, 39].

**Fig. (1):**
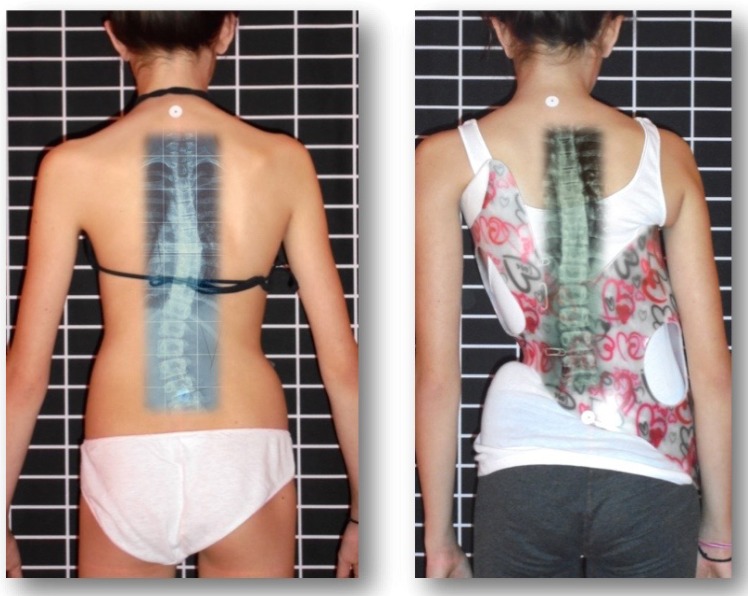
In-brace correction of a double curve pattern. More than 60% correction can be achieved in the Gensingen brace (GBW) when the curve is still flexible.

The purpose of this study is to evaluate a sample of patients with Cobb angles of *≥*40 degrees treated with the Gensingen Brace (a Cheneau-style TLSO) (Fig. **1**), and to determine whether brace treatment in curvatures of *≥*40 degrees can be successful. The cohort of this prospective study complies with all the SRS inclusion criteria for bracing [46] with the one exception. The degree of Cobb angle is larger in this population, but includes immature patients at high risk of progression, so that our results can be compared to the results of BrAIST [37]. For patients who decline surgery, studies such as ours will help establish the benefits and/or disadvantages of bracing severe scoliosis and help patients make informed decisions about how to proceed with treatment options.

Ethical considerations: There is no high-level evidence indicating that spinal fusion surgery is superior to natural history [9, 14, 20-25]. Therefore, per the recent suggestions made by Ward and colleagues [30, 31], other treatment options for patients with curvatures *≥*40° should be investigated.

## 2 MATERIALS AND METHODS

### 2.1 Patient Population

Twenty-five female patients (Risser 0-2) were included in this report. With the exception of having a *≥*40° Cobb angle, all patients satisfied the SRS inclusion criteria. Patients were fit with the Gensingen brace (GBW) at a bracing facility affiliated with the first author’s clinic. All patients were followed prospectively for a minimum of 18 months, with an average follow-up time of 30.4 months (SD 9.2) and with an average x-ray follow-up of 20 months (SD 9.4). The average curvature at the start of treatment was 49 degrees (SD 8.4; 40 – 71º) (12 double and 13 single patterns of curvature). The average age was 12.4 years (SD 0.82), average Risser was 0.84 (SD 0.94), and fourteen out of the twenty-five females were pre-menarcheal.

### 2.2 Brace Development Process

**Fig. (2):**
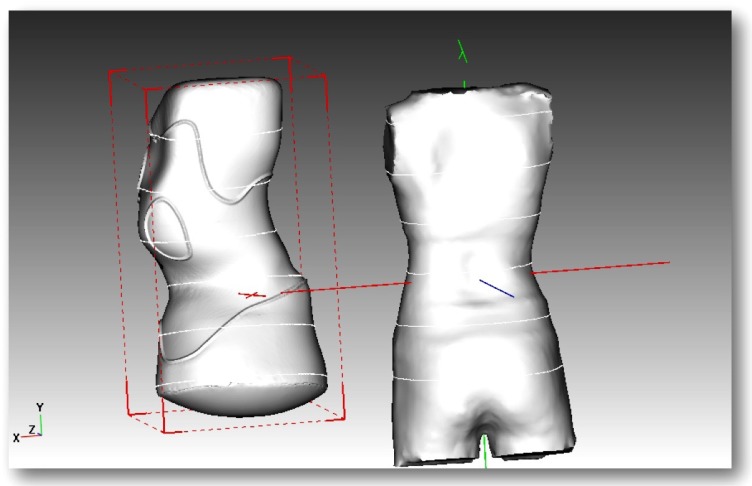
CAD modeling of a GBW for a single thoracic curve. Mirroring of the deformity (patient’s scan on the right) in the brace model (left) is clearly visible.

**Fig. (3):**
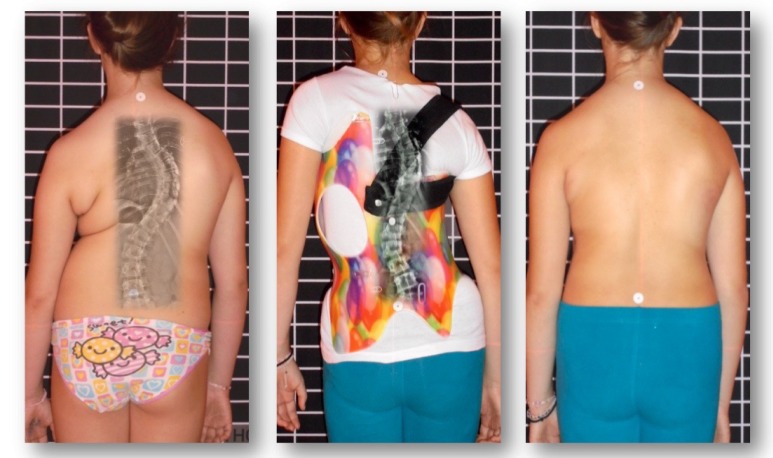
In-brace correction of a single thoracic curve pattern exceeding 70° in the GBW. The follow-up (right) shows the curve is rebalanced and that postural improvement has been achieved, despite the severity of the initial Cobb angle and noticeable asymmetries.

The Gensingen brace is the result of the recent advancements of Chêneau principles [42] and was first described in 2010 [41]. Each orthosis is made via computer-aided-design (CAD) (Fig. **2**). Each GBW is based on the augmented Lehnert-Schroth (ALS) classification system [4, 42] and is pattern-specific based on a patient’s 3D scan, x-ray, scoliometer measurements, and postural assessment. There are seven basic brace models corresponding to the ALS classification pattern and two additional models for larger curves [42]. The additional brace models for single thoracic curves exceeding 60° have been developed (Fig. **3**) because of an increasing number of patients with higher Cobb angles who are seeking conservative treatment.

### 2.3 X-rays and Follow-Up

X-rays were done prior to the start of treatment, in the brace, before and after each subsequent brace and at skeletal maturity (after brace weaning). If there were clinical signs of deterioration, additional x-rays were taken as well. For local patients, in-brace x-rays were taken 6 weeks after the start of brace-wear. For patients visiting from a distance, in-brace x-rays were taken the following day after fitting. The average in-brace Cobb angle measured 28.5° (SD 14.7; 42% correction). It was recommended that patients wear their brace for 20 hours per day or more; however, the braces did not include any sensors to monitor wear-time. It should be noted that there is a potential for bias as all Cobb angle measurements were done by the senior author.

### 2.4 Statistical Analysis

A z-test to compare cohorts of different sizes, as proposed by Goldberg [43], was performed to compare the success rate of this cohort to the success rate of patients from the BrAIST cohort, a study which predominately used the Boston-type brace (68% of braced patients) [37]. In the BrAIST study, 146 patients were braced and followed through skeletal maturity, while in our sample 25 patients have been treated and followed prospectively for a minimum of 18 months. In our study, halted progression and curve improvement (decrease of 6 degrees or more) were considered treatment success, while curve progression was considered treatment failure (increase of 6 degrees or more). The first author also measured each patient’s thoracic and lumbar Angle of Trunk Rotation (ATR) before treatment and at last follow-up. A paired sample t-test was then performed to determine if the differences were statistically significant.

## 3 RESULTS

The average Cobb angle after follow-up was 44.2 degrees (SD 12.9). Two of the twenty-five patients progressed (curve increased 6 degrees or more) while eleven patients improved (curve decreased 6 degrees or more) and twelve patients were able to halt progression (curve remained within 5 degree margin of error). When comparing the BrAIST cohort to this GBW cohort, the differences in the success rate (72% and 92%, respectively) were statistically significant in the z-test (z = -3.041; p = 0.01). Additionally, in the thoracic spine, average ATR decreased from 12.2 to 10.1 degrees (p = 0.11) and in the lumbar spine, average ATR decreased from 4.7 to 3.6 degrees (p = 0.0074).

The average clinical follow-up time was 30.4 months (SD 9.2) and the average radiological follow-up was 20 months (SD 9.5). Patients in the cohort reported an average brace-wearing time of 21 hours per day, before the weaning-off phase when they were instructed to reduce their wearing hours. By the end of the current study, six patients had completely weaned off the brace, with four of the six having shown improvements of *≥*6°, and the other two being stable (+/-5 degrees).

**Fig. (4):**
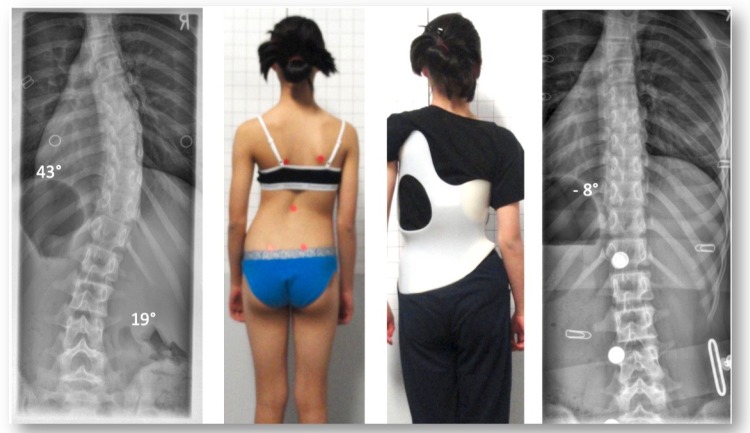
A 12-year old skeletally immature girl from New Zealand with a single thoracic curve of 43° and an overcorrection in the GBW (model 2012) to -8° [42].

**Fig. (5):**
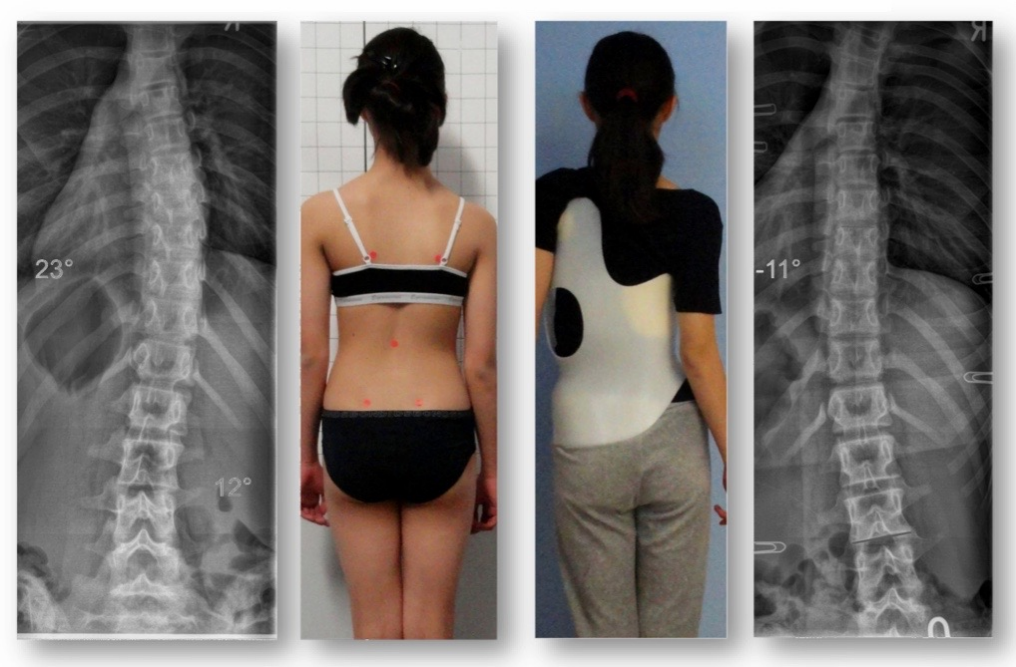
Intermediate result of the girl from Fig. (**4**) after 6 months of full-time treatment. At that time, she had outgrown her first brace and a second brace was made. As shown in the x-ray on the right, she achieved overcorrection in her second brace as well.

**Fig. (6):**
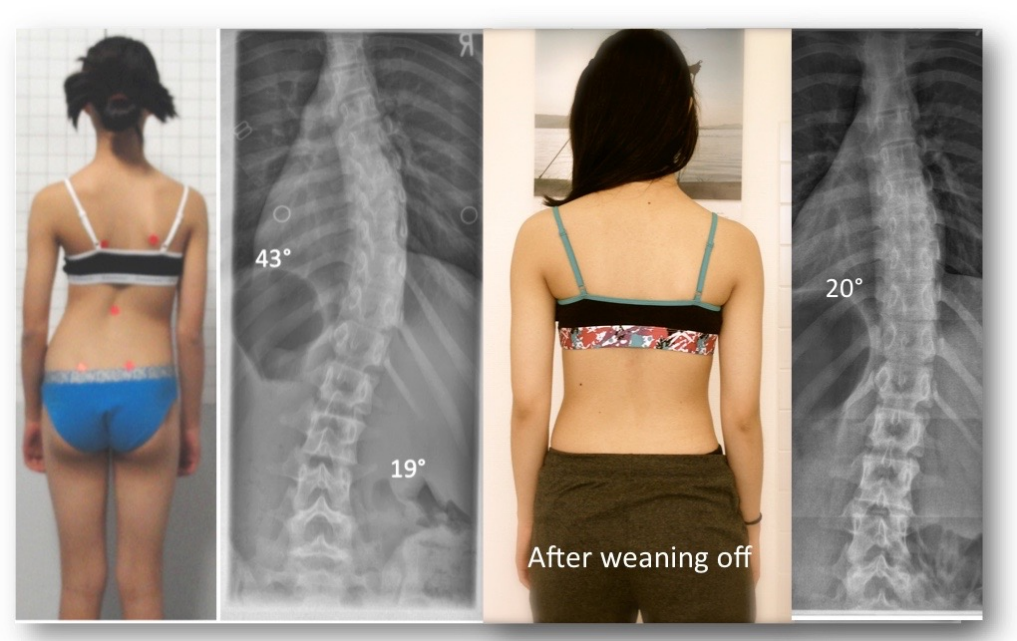
The patient weaned off the brace in the summer of 2014. At skeletal maturity, her Cobb angle measured 20° and she had a more compensated posture in comparison to her initial posture (left).

One of the four patients who experienced curve improvement was a girl from New Zealand. She initially began treatment at age 12, with a 43° Cobb angle (Risser 0, Tanner II, premenarcheal). At skeletal maturity (Risser 5, age 16), her Cobb angle measured 20°. During the course of treatment she regularly followed up at the office of the first author, in Germany, and after three years of treatment, she was successfully weaned off her second Gensingen brace in summer 2014 (Figs. **4**, **5** and **6**). The patient plans to have a new x-ray taken when she completes high school, however this >2-year post-weaning x-ray is not yet available.

One patient dropped out and was not included in this cohort. This patient presented with a double major curve pattern of >50°. Over the course of 2 years, she was fit with two Gensingen braces at the facility of the first author and her curves were stable. When the patient needed a third brace, her mother decided to try an orthotist closer to their home for an alternative Chêneau-style brace. The patient’s curves eventually progressed to more than 75° and she returned to the first author for another Gensingen brace, since she had declined surgery. At that point, we were unable to improve her curve, but cosmetically the deformity was not very obvious.

## 4 DISCUSSION

Different brace types lead to different outcomes. It has been determined that symmetric braces (with asymmetric pads only) are effective in 70-72% of the cases when the SRS inclusion criteria are respected [36, 37]. Asymmetric scoliosis braces differ in several ways and have demonstrated an even higher rate of success [32, 33, 38-40].

Although the success rate of asymmetric braces can vary significantly, this is likely attributable to how the brace is designed, manufactured and fitted. When manufactured by hand, on the basis of a plaster cast, success rates are between 48% [44] and 80% [33] among comparable groups. This large discrepancy suggests that the success of scoliosis bracing is at least partially influenced by the experience and skills of the brace technician.

The Gensingen brace used in this study was produced with CAD/CAM technology, which allows for standardization [42]. For any given curve pattern, the basic brace model is the same, which reduces the risk for human error. While the brace can be created with measurements of the patient’s trunk at certain anatomical landmarks, most Gensingen braces currently made worldwide are designed from a patient’s 3D scan. Using the scan, the virtual brace model is further individualized with the addition of correction forces in all three planes. A final STL-file is then sent for manufacturing.

Additionally, while many brace models work by pushing against the prominences and convexities of the curvature(s), the objective of the Gensingen brace is to implement a corrective movement as well [42]. In order to avoid compression of the trunk, voids are implemented opposite the pressure zones so that curve improvement is only limited by the stiffness of the patient’s spine. These distinctions are integral to the design of the Gensingen brace and for that reason the results of this study cannot be extrapolated to other braces.

When comparing the results from this study (92% success rate) to the results achieved with the Boston brace (72% success rate), it is important to note that the two studies had a different definition of treatment success and failure. In the study by Weinstein and colleagues, patients started with a Cobb angle of 25°-40° and treatment was considered successful when the curve did not exceed 49° [37]. This means that their scoliosis could progress significantly, but still be labeled a success as long as it did not reach 50°. In our study, patients whose Cobb angle progressed 6° or more were labeled as treatment failure, regardless of their initial Cobb angle. All things being equal, if the BrAIST study had used these stricter parameters, it is likely that their reported success rate would have been adversely affected.

**Fig. (7):**
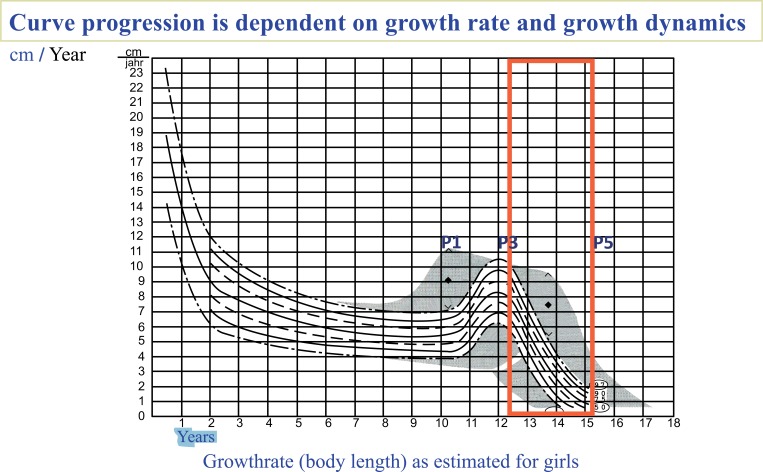
The average starting age for patients in the study was 12.4 years. After an average follow-up time of 30.4 months (see red frame), the patients are more mature, their growth rate is decreased and the risk of further progression is significantly reduced.

Our initial results are encouraging, especially when considering the fact that the patients included in our sample were at high risk for progression with respect to maturity and severity of scoliosis [47]. According to Lonstein and Carlson [47], the average progression factor for this cohort was 4 – meaning that the risk of progression for the average patient was 100%. At the start of the study, the average patient age was 12.4 years – an age during which an adolescent typically starts to enter the descendent phase of the pubertal growth spurt (Fig. **7**). Though some patients were still wearing or weaning off the brace at the end of the study, after the average 30.4 month-long follow-up period their growth spurt was nearly complete, corresponding to a lower risk of progression (Fig. **7**).

While the results presented in this paper can only be regarded as preliminary, as our cohort continues to mature, the risk for progression is far less than at the start of the observation period. We will continue to monitor the results of the study participants until all twenty-five have completed treatment and have discontinued brace-wear. Additional studies are needed to validate the use of highly corrective asymmetric braces as a viable non-surgical alternative for skeletally immature patients who are willing to comply with conservative treatment.

## CONCLUSION

Conservative brace treatment using the Gensingen brace was successful in 92% of cases of patients with AIS of 40 degrees and higher. This is a significant improvement compared to the results attained in the BrAIST study (72%). Reduction of the ATR shows that postural improvement is also possible.
